# Synthesis, crystal structure and Hirshfeld surface analysis of [bis­(di­phenyl­phosphan­yl)methane-κ*P*]chloridobis­[2-(pyridin-2-yl)phenyl-κ^2^ *N*,*C* ^1^]iridium(III)

**DOI:** 10.1107/S2056989021000955

**Published:** 2021-02-02

**Authors:** Ekkapong Klaimanee, Peerapong Sangwisut, Saowanit Saithong, Nararak Leesakul

**Affiliations:** a Division of Physical Science and Center of Excellence for Innovation in Chemistry, Faculty of Science, Prince of Songkla University, Hat-Yai, Songkhla, 90112, Thailand

**Keywords:** crystal structure, iridium, 2-phenyl­pyridine, di­phenyl­phosphanyl­methane

## Abstract

The title Ir^III^ complex, [Ir(C_11_H_8_N)_2_Cl(C_25_H_22_P_2_)], was synthesized from the substitution reaction between the (ppy)_2_Ir(μ-Cl)_2_Ir(ppy)_2_ (ppy = deprotonated 2-phenyl­pyridine, C_11_H_8_N^−^) dimer and 1,1-bis­(di­phenyl­phosphan­yl)methane (dppm, C_25_H_22_P_2_) under an argon gas atmosphere for 20 h. The Ir^III^ atom is coordinated by two C,*N*-bidentate ppy anions, a unidentate dppm ligand and a chloride anion in a distorted octa­hedral IrC_2_N_2_PCl arrangement. The N donor atoms of the ppy ligands are mutually *trans* while the C atoms are *cis*. Intra­molecular aromatic π–π stacking between the phenyl rings of ppy and dppm, and C—H⋯Cl inter­actions are observed. In the crystal, C—H⋯Cl and C—H⋯π contacts link the mol­ecules into a three-dimensional network. A Hirshfeld surface analysis was carried out to further qu­antify the inter­molecular inter­actions, and indicated that H⋯H contacts (63.9%) dominate the packing.

## Abstract 2

The title Ir^III^ complex was synthesized from the substitution reaction between the (ppy)_2_Ir(μ-Cl)_2_Ir(ppy)_2_ (ppy = deprotonated 2-phenyl­pyridine, C_11_H_8_N^−^) dimer and 1,1-bis­(di­phenyl­phosphan­yl)methane (dppm, C_25_H_22_P_2_) under an argon gas atmosphere. The Ir^III^ atom is coordinated by two C,*N*-bidentate ppy anions, a unidentate dppm ligand and a chloride anion in a distorted octa­hedral IrC_2_N_2_PCl arrangement.

## Chemical context   

Iridium(III) complexes have been investigated for decades because of their stability (Jian *et al.*, 2011 ▸; Lee *et al.*, 2009 ▸; Tsuboyama *et al.*, 2003 ▸), promising luminescent properties (Lin *et al.*, 2011 ▸; Lowry *et al.*, 2004 ▸; Tamayo *et al.*, 2003 ▸) and medicinal applications, especially as anti­cancer agents (Hearn *et al.*, 2018 ▸; Rubio *et al.*, 2020 ▸; Xiao *et al.*, 2018 ▸). The syntheses of cyclo­metallated iridium(III) complexes have mainly focused on the 2-phenyl­pyridine (ppy) ligand and its derivatives. The octa­hedral geometry of bis-complexes is commonly selected as the main backbone accompanied by various types of ancillary ligands. Most of them are N-donor ligands (Chi & Chou, 2010 ▸; Goldsmith *et al.*, 2005 ▸; Lin *et al.*, 2011 ▸) owing to the strong binding of the borderline acid metal and basic ligand. However, there are fewer reports of P-donor ancillary ligands. In this present work, we report the synthesis and characterization of the title photoactive complex, (I), obtained by the reaction between (ppy)_2_Ir(μ-Cl)_2_Ir(ppy)_2_) dimer (ppy = deprotonated 2-phenyl­pyridine, C_11_H_8_N^−^) with 1,1-bis­(di­phenyl­phosphan­yl)methane under an inert gas atmosphere.
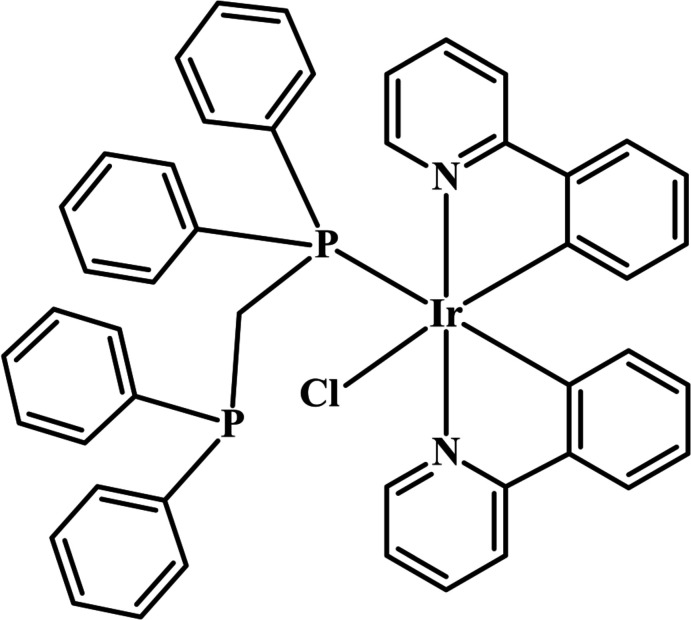


## Structural commentary   

**Figure 1:**
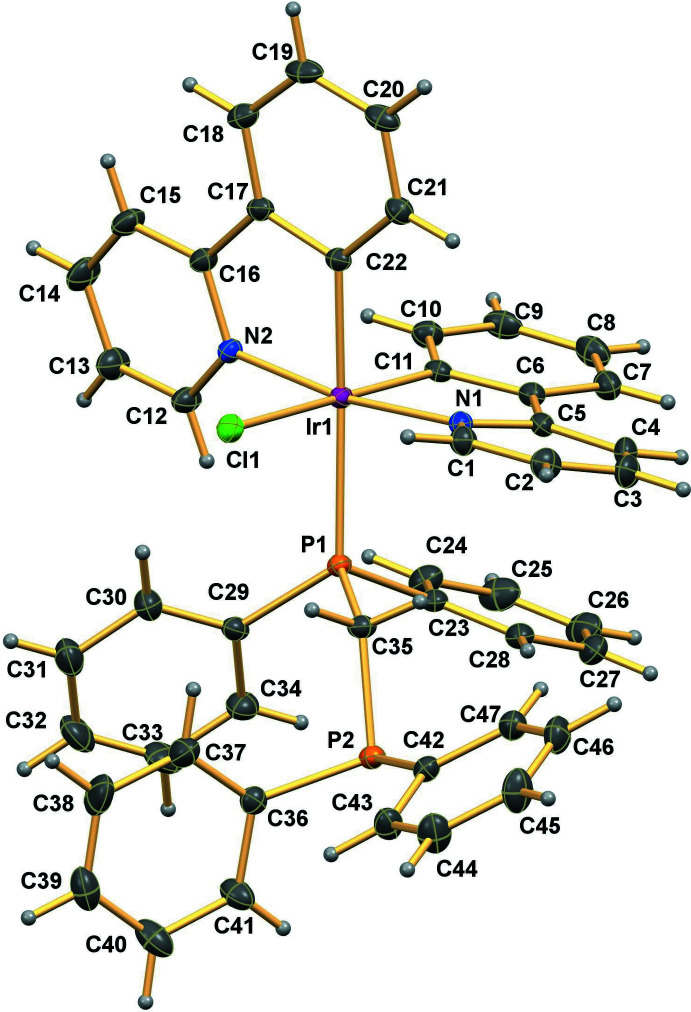
The mol­ecular structure of the title compound, including atom labelling. Displacement ellipsoids are drawn at the 30% probability level.

The asymmetric unit of (I) shows a distorted octa­hedral mol­ecular structure to overcome steric hindrance between the ligands (Fig. 1 ▸) in space group *P*2_1_/*n*. The Ir^III^ atom is linked to two C,N-bidentate 2-phenyl­pyridine (ppy) anions through five-membered chelate rings where the N1 and N2 atoms of the ppy pyridine rings exist in a *trans* orientation to each other [N1—Ir1—N2 = 170.97 (9)°] and C11 and C22 are in *cis* orientation [C11—Ir1—C22 = 91.12 (11)°]. The bond lengths of Ir1—N1, Ir1—N2, Ir1—C11 and Ir1—C22 are 2.051 (2), 2.062 (2), 2.004 (3) and 2.032 (3) Å, respectively. As expected, the averaged Ir–C and Ir—N bond lengths are much shorter than the Ir—Cl and Ir—P bonds, based on the sizes of the different species.

The averaged Ir—N and Ir—C distances in (I) are both slightly shorter than those in [Ir(ppy)_2_(dppm)]PF_6_ (Hao *et al.*, 2019 ▸). However, the averaged Ir—N distance is a little longer, but the Ir—C bond lengths are relatively shorter than those of related Ir^III^ complexes bonded with ppy ligands (Chen *et al.*, 2015 ▸; Shen *et al.*, 2011 ▸; Wang *et al.*, 2005 ▸)

Although bis­(di­phenyl­phosphan­yl)methane (dppm) often occurs as a bidentate ligand (*e.g.*, Hao *et al.*, 2019 ▸), in (I) it is unidentate [Ir1—P1 = 2.4241 (7) Å]. This Ir—P distance is somewhat longer than that in the [Ir(ppy)_2_(dppm)](PF_6_) (Hao *et al.*, 2019 ▸) complex. The Ir—Cl bond distances in chloro­bis­[2-(2-pyrid­yl)phenyl-κ^2^ *N*,*C*](tri­phenyl­phosphine-κ*P*)iridium(III) are reported to be 2.503 (19) (Wang *et al.*, 2005 ▸) and 2.505 (16) (Shen *et al.*, 2011 ▸) Å, which are slightly longer than that in (I) [Ir1—Cl1 = 2.4866 (8) Å]. The *cis* bond angles in (I) all deviate from the ideal value of 90° [80.07 (10)–95.27 (7)°] and likewise, the *trans* bond angles deviate from the ideal 180° [170.97 (9)–175.37 (8)°], similar to related compounds (Shen *et al.*, 2011 ▸; Wang *et al.*, 2005 ▸). The dihedral angle between the mean planes of the ppy rings is 77.98 (4)°, indicating the *cis*-form of the chelate rings. Key geometrical data are given in Table 1 ▸.

**Figure 2:**
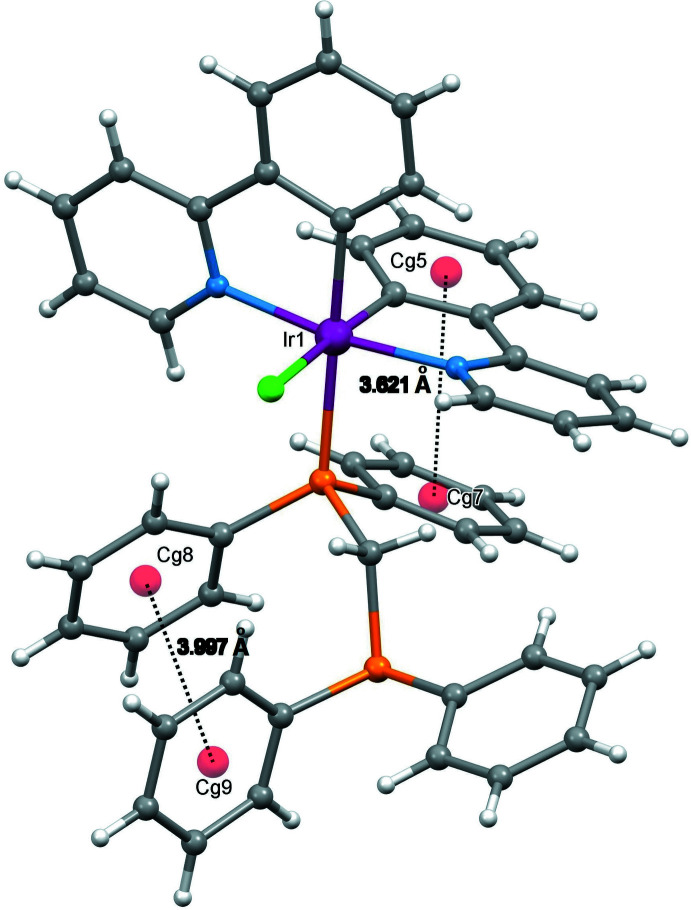
Intra­molecular π–π inter­actions occurred between the phenyl rings of the complex (H atoms are omitted).

**Figure 3:**
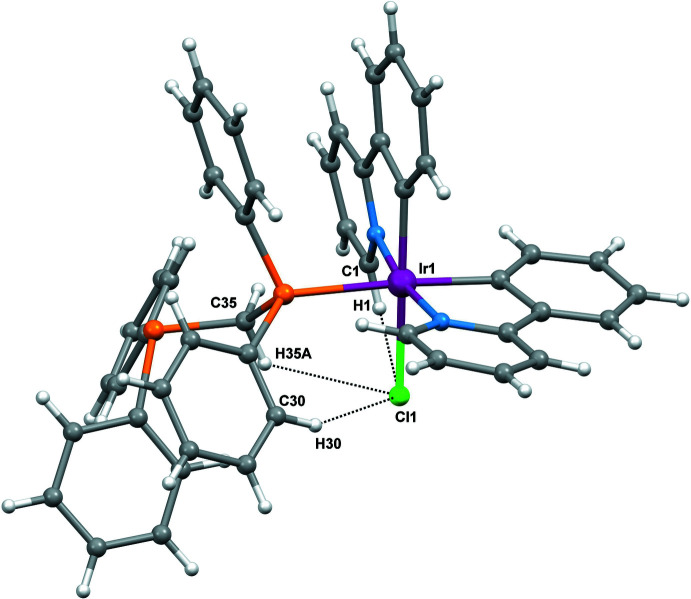
The intra­molecular C—H⋯Cl inter­actions in the title compound

Intra­molecular π–π stacking inter­actions are observed for (I). The π–π stackings are found between two phenyl rings (C6–C11 and C23–C28) of the ppy and dppm ligands, *Cg*5⋯*Cg*7 = 3.621 (1) Å and between the phenyl rings of the dppm mol­ecule (C29–C34 and C36–C41), *Cg*8⋯*Cg*9 = 3.997 (1) Å (Fig. 2 ▸). Three weak intra­molecular hydrogen-bonding inter­actions, *viz*. C1—H1⋯Cl1 [C⋯Cl = 3.357 (3) Å], C30—H30⋯Cl1 [C⋯Cl = 3.664 (4) Å] and C35—H35*A*⋯Cl1 [C35⋯Cl = 3.460 (3) Å] (Fig. 3 ▸ and Table 2 ▸) are observed.

## Supra­molecular features   

**Figure 4:**
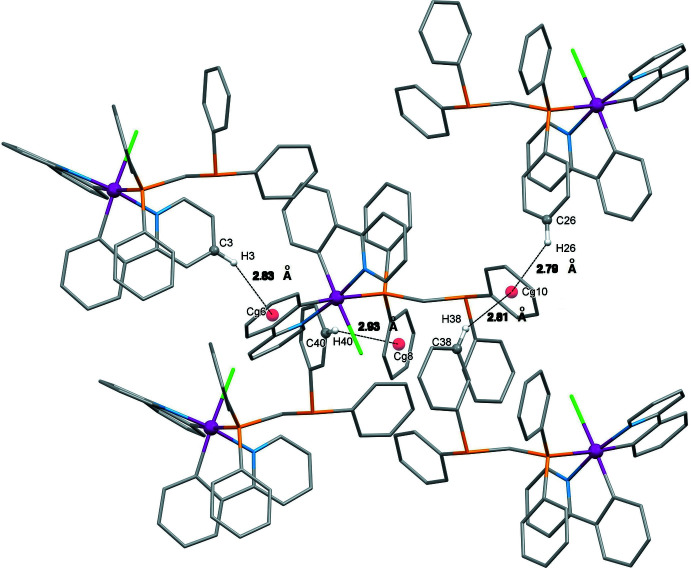
The inter­molecular C—H**⋯·**π inter­actions in the title compound.

Several weak C—H**⋯**π (ring) inter­actions are found in the crystal packing (Fig. 4 ▸). The inter­actions are observed between any two adjacent mol­ecules of ppy *via* the C3—H3 grouping of the pyridine ring and the centroid (*Cg*6) of the C17–C22 phenyl ring (H3⋯*Cg*6 = 2.83 Å). In addition, C—H⋯π(ring) inter­actions are also found between the dppm phenyl rings of neighbouring mol­ecules: C26—H26 ⋯*Cg*10 (H26⋯*Cg*10 = 2.79 Å; *Cg*10 is the centroid of the C42–C47 ring), C38—H38⋯*Cg*10 (H38⋯*Cg*10 = 2.81 Å) and C40—H40⋯*Cg*8 (H40⋯*Cg*8 = 2.95 Å). In addition, pairwise inter­molecular hydrogen bonds are observed between C14—H14 of the pyridine ring of the ppy ring and Cl1 (Table 2 ▸).

## Hirshfeld surface analysis   

**Figure 5:**
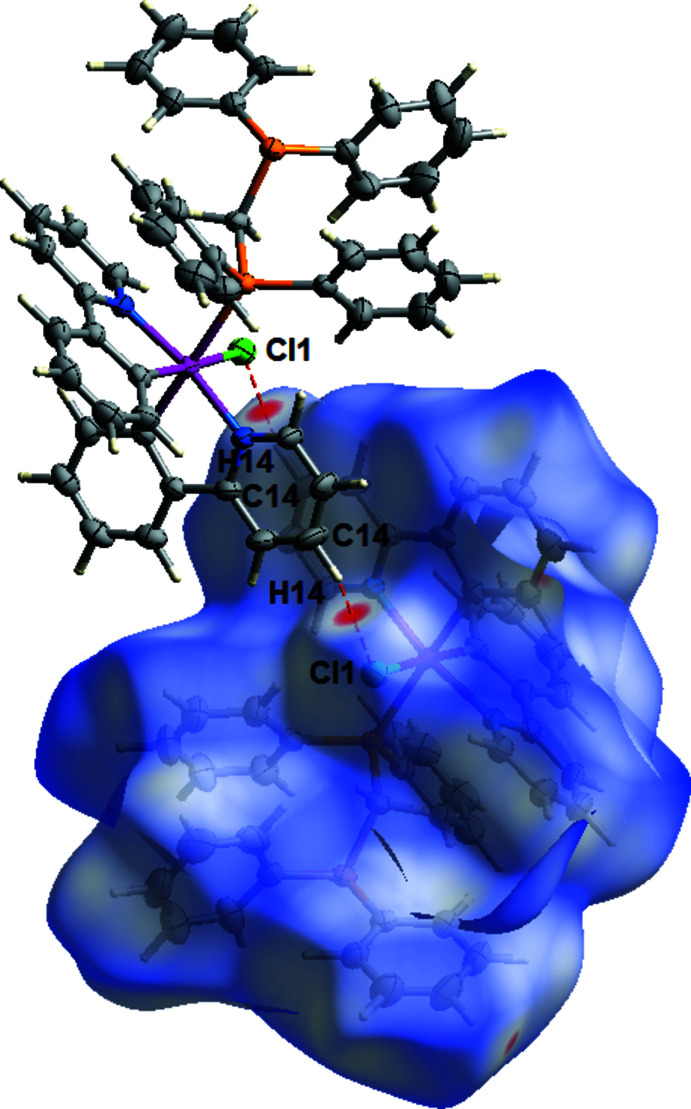
Hirshfeld surface plot showing the C—H**⋯·**Cl inter­actions.

**Figure 6:**
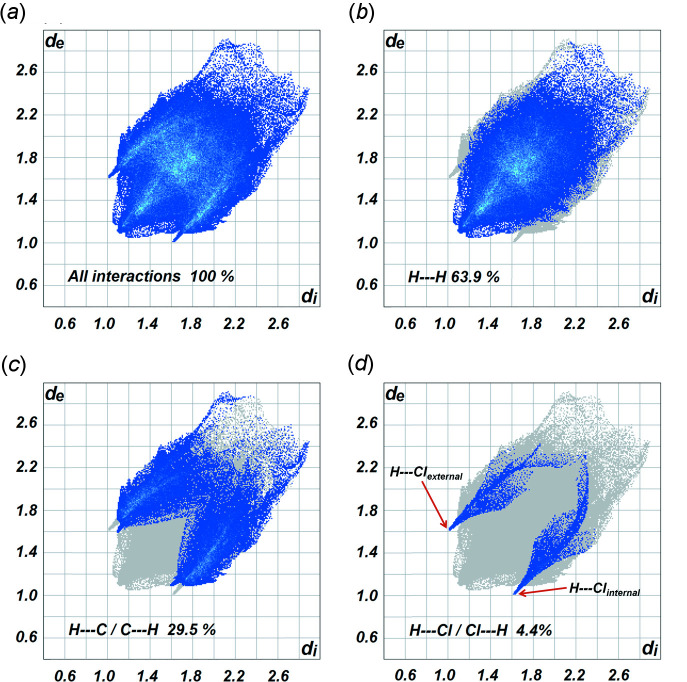
Fingerprint plots corresponding to inter­molecular contacts in the crystal: (*a*) all inter­actions, (*b*) H⋯H contacts, (*c*) H⋯C/C⋯H and (*d*) H⋯Cl/Cl⋯H.

Additional insights into the weak inter­molecular contacts in the crystal packing of (I) were gained from Hirshfeld surface analysis and the two-dimensional fingerprint plots (McKinnon *et al.*, 2004 ▸; 2007 ▸; Spackman & Jayatilaka, 2009 ▸) generated using *Crystal Explorer 17.5* program (Turner *et al.*, 2017 ▸). The Hirshfeld surfaces were mapped over the normalized contact distance (*d*_norm_) with the functions *d*_e_ and *d*_i_, which are the distances from an indicated area on the Hirshfeld surface to the nearest atoms outside and inside the surface, respectively. The white, red, and blue areas on the *d*_norm_-mapped Hirshfeld surfaces show inter­molecular contacts that are equal to, shorter than, and longer than the sum of their van der Waals (vdW) radii, respectively. A pair of inter­molecular contacts are shown as red spots on the Hirshfeld surface close to the Cl1 atom of the adjoining mol­ecule and the H14 atom of the associated pyridine ring. The spots indicate hydrogen-bond donor-to-acceptor inter­actions of C14—H14⋯Cl1 and *vice versa* (Fig. 5 ▸). The relative contributions of the various types of contacts to the total of inter­molecular inter­actions across the Hirshfeld surface are represented in two-dimensional fingerprint plots. Total inter­molecular inter­actions (100%) are shown in Fig. 6 ▸(*a*) while Fig. 6 ▸(*b*)–(*d*) depict the contacts of the H⋯H (63.9%), C⋯H/H⋯C (29.5%) and H⋯Cl/Cl⋯H (4.4%) inter­actions, respectively.

## Database survey   

A search of the *SciFinder* (SciFinder, 2020 ▸) database for phospho­rescent complexes of ppy with iridium(III) diphos­phine (dpp) reveals eight structures closely related to the title compound. Hao *et al.* (2019 ▸) report the crystal structure of an ionic complex of the [Ir(ppy)_2_(dppm)]^+^; dppm = bis­(di­phenyl­phosphan­yl)methane bidentate ligand. However, none of the remaining publications describe a monomeric Ir^III^ complex similar to the title compound. The seven hits include the octa­hedral crystal structures of Ir^III^ complexes with a bis­(2-phenyl­pyridine)­iridium(III) backbone and ancillary ligands of both N-donor, P-donor and O-donor ligands. There are a *tris*-complex of Ir(ppy)_3_ (Huynh *et al.*, 2005 ▸), [Ir(ppy)_2_(dppel)]; dppel = 1,2-bis­(di­phenyl­phosphan­yl)ethyl­ene, [Ir(ppy)_2_(dppp)]; dppp = 1,3-bis­(di­phenyl­phosphan­yl)propane and [Ir(ppy)_2_(dppe)]; dppe = 1,2-bis­(diphenylphosphan­yl) ethane] (Alam *et al.*, 2013 ▸), [Ir(ppy)_2_ *L*_2_]^+^ (*L*_2_ = substituted 2,2′-bi­pyridine, dppe and 1,10-phenanthroline; Lowry & Bernhard, 2006 ▸), [Ir(ppy)_2_(P^N)]PF_6_, [Ir(dfppy)_2_(P^N)]PF_6_ and [Ir(dfmppy)_2_(P^N)]PF_6_ where P^N = 2-[(di­phenyl­phos­phan­yl) meth­yl]pyridine, dfppy = 2-(2,4-difuorophen­yl)pyri­dine and dfmppy = 2-(2,4-di­fluoro­phen­yl)-4-methyl­pyridine (Ma *et al.*, 2009 ▸), [Ir(ppy)_2_(biq)]PF_6_ (biq = 2,2-bi­quinoline; Nishikitani *et al.*, 2018 ▸) and Ir(dppy)_2_(acac) (dppy = 2,5-di­phenyl­pyridyl and acac = acetyl­acetonate; Xu *et al.*, 2005 ▸). There are four other related complexes, Ir(ppy)_2_(*L*) [*L* = 1,2-bis­(di­phenyl­phosphan­yl)ethane, 1,2-bis (di­phenyl­phosphan­yl)propane, 1,2-bis­(di­phenyl­phos phino)benzene and 1,8-bis­(di­phenyl­phos phino)naphthalene; Liu *et al.*, 2019 ▸; Luo *et al.*, 2013 ▸] and Ir(ppy)_2_(PPh_3_)Cl (Wang *et al.*, 2005 ▸).

## Synthesis and crystallization   

The title complex was synthesized from the reaction between (ppy)_2_Ir(μ-Cl)2Ir(ppy)_2_ (0.5 mmol) and bis­(di­phenyl­phosphan­yl)methane (1.25 mmol) in CH_2_Cl_2_ solution. The reaction was carried out by refluxing the mixture under Ar gas for 20 h. The solution mixture was then cooled to room temperature and the solvent was evaporated. The crude yellow product thus obtained was washed with diethyl ether to remove excess ligands and impurities, and the complex was crystallized and recrystallized in mixed solvents of di­chloro­methane:diethyl ether (9:1) at room temperature three times, yielding yellowish crystals (yield = 30%), m.p. = 488–489 K IR (KBr, cm^−1^): ν(C—H), 3054; ν(C=C), 1436, 2367; ν(C—N), 1030; ν(C=N), 1613; ν(P—Ph), 1098; ν(Ir—P), 760; ν(Ir—N), 733; ν(Ir—Cl), 510. Analysis (%): found C 61.01, H 4.38, N 2.77; calculated C 61.33, H 4.16, N 3.04.

## Refinement   

Crystal data, data collection and structure refinement details are summarized in Table 3 ▸. H atoms were included in calculated positions [C—H = 0.93 (aromatic) or 0.97 Å (C*sp^2^*)] and refined as riding with *U*_iso_(H) = 1.2*U*_eq_(C).

## Supplementary Material

Crystal structure: contains datablock(s) I. DOI: 10.1107/S2056989021000955/hb7961sup1.cif

Structure factors: contains datablock(s) I. DOI: 10.1107/S2056989021000955/hb7961Isup2.hkl

Supporting information file. DOI: 10.1107/S2056989021000955/hb7961Isup3.mol

CCDC reference: 2058995

Additional supporting information:  crystallographic information; 3D view; checkCIF report

## Figures and Tables

**Table 1:** Selected geometric parameters (Å, °)

Ir1—C11	2.004 (3)	Ir1—N2	2.062 (2)
Ir1—C22	2.032 (3)	Ir1—P1	2.4241 (7)
Ir1—N1	2.051 (2)	Ir1—Cl1	2.4866 (8)
			
C11—Ir1—C22	91.12 (11)	N1—Ir1—P1	85.83 (6)
C11—Ir1—N1	80.35 (10)	N2—Ir1—P1	101.93 (6)
C22—Ir1—N1	92.62 (10)	C11—Ir1—Cl1	175.37 (8)
C11—Ir1—N2	94.40 (10)	C22—Ir1—Cl1	87.54 (8)
C22—Ir1—N2	80.07 (10)	N1—Ir1—Cl1	95.27 (7)
N1—Ir1—N2	170.97 (9)	N2—Ir1—Cl1	89.74 (7)
C11—Ir1—P1	93.75 (8)	P1—Ir1—Cl1	87.41 (3)
C22—Ir1—P1	174.57 (8)		

**Table 2:** Hydrogen-bond geometry (Å, °) *Cg*6, *Cg*8 and *Cg*10 are the centroids of the C17–C22, C29–C34 and C42–C47 rings, respectively.

*D*—H⋯*A*	*D*—H	H⋯*A*	*D*⋯*A*	*D*—H⋯*A*
C1—H1⋯Cl1	0.93	2.73	3.357 (3)	126
C14—H14⋯Cl1^i^	0.93	2.77	3.548 (4)	142
C30—H30⋯Cl1	0.93	2.84	3.664 (4)	148
C35—H35*A*⋯Cl1	0.97	2.83	3.460 (3)	124
C3—H3⋯*Cg*6^ii^	0.93	2.83	3.572 (4)	137
C26—H26⋯*Cg*10^iii^	0.93	2.79	3.575 (5)	143
C38—H38⋯C10^iii^	0.93	2.81	3.659 (5)	153
C40—H40⋯*Cg*8^iv^	0.93	2.95	3.711 (5)	140

Symmetry codes: (i) -x+1, -y, -z+2; (ii) -x+{\script{3\over 2}}, y+{\script{1\over 2}}, -z+{\script{3\over 2}}; (iii) -x+2, -y+1, -z+2; (iv) -x+{\script{3\over 2}}, y+{\script{1\over 2}}, -z+{\script{5\over 2}}.

**Table 3:** Experimental details

Crystal data	
Chemical formula	[Ir(C_11_H_8_N)_2_Cl(C_25_H_22_P_2_)]
*M*_r_	920.38
Crystal system, space group	Monoclinic, *P*2_1_/*n*
Temperature (K)	293
*a*, *b*, *c* (Å)	14.4506 (5), 15.4490 (5), 17.8532 (6)
β (°)	103.044 (1)
*V* (Å^3^)	3882.8 (2)
*Z*	4
Radiation type	Mo *K*α
μ (mm^−1^)	3.63
Crystal size (mm)	0.19 × 0.09 × 0.06

Data collection	
Diffractometer	Bruker *APEX* CCD area-detector
Absorption correction	Multi-scan (*SADABS*; Bruker, 2003 ▸)
*T*_min_, *T*_max_	0.800, 1.000
No. of measured, independent and observed [*I* > 2σ(*I*)] reflections	35153, 9248, 7758
*R*_int_	0.033
(sin θ/λ)_max_ (Å^−1^)	0.658

Refinement	
*R*[*F* ^2^ > 2σ(*F* ^2^)], *wR*(*F* ^2^), *S*	0.026, 0.062, 1.05
No. of reflections	9248
No. of parameters	478
H-atom treatment	H-atom parameters constrained
Δρ_max_, Δρ_min_ (e Å^−3^)	0.90, −0.33

Computer programs: *SMART* and *SAINT* (Bruker, 2003 ▸), *SHELXT2014* (Sheldrick, 2015*a* ▸), *ShelXle* (Hübschle *et al.*, 2011 ▸), *SHELXL2014/7* (Sheldrick, 2015*b* ▸), *Mercury* (Macrae *et al.*, 2020 ▸), *WinGX* publication routines (Farrugia, 2012 ▸) and *publCIF* (Westrip, 2010 ▸).

## supplementary crystallographic information

### Crystal data

**Table:**

[Ir(C_11_H_8_N)_2_Cl(C_25_H_22_P_2_)]	*F*(000) = 1832
*M**_r_* = 920.38	*D*_x_ = 1.574 Mg m^−^^3^
Monoclinic, *P*2_1_/*n*	Mo *K*α radiation, λ = 0.71073 Å
*a* = 14.4506 (5) Å	Cell parameters from 9441 reflections
*b* = 15.4490 (5) Å	θ = 2.3–25.6°
*c* = 17.8532 (6) Å	µ = 3.63 mm^−^^1^
β = 103.044 (1)°	*T* = 293 K
*V* = 3882.8 (2) Å^3^	Block, light yellow
*Z* = 4	0.19 × 0.09 × 0.06 mm

### Data collection

**Table:**

Bruker APEX CCD area-detector diffractometer	9248 independent reflections
Radiation source: fine-focus sealed tube	7758 reflections with *I* > 2σ(*I*)
Graphite monochromator	*R*_int_ = 0.033
Frames, each covering 0.3 ° in ω scans	θ_max_ = 27.9°, θ_min_ = 1.6°
Absorption correction: multi-scan (SADABS; Bruker, 2003)	*h* = −19→18
*T*_min_ = 0.800, *T*_max_ = 1.000	*k* = −20→20
35153 measured reflections	*l* = −23→23

### Refinement

**Table:**

Refinement on *F*^2^	Primary atom site location: structure-invariant direct methods
Least-squares matrix: full	Secondary atom site location: difference Fourier map
*R*[*F*^2^ > 2σ(*F*^2^)] = 0.026	Hydrogen site location: inferred from neighbouring sites
*wR*(*F*^2^) = 0.062	H-atom parameters constrained
*S* = 1.05	*w* = 1/[σ^2^(*F*_o_^2^) + (0.0298*P*)^2^ + 0.328*P*] where *P* = (*F*_o_^2^ + 2*F*_c_^2^)/3
9248 reflections	(Δ/σ)_max_ = 0.004
478 parameters	Δρ_max_ = 0.90 e Å^−^^3^
0 restraints	Δρ_min_ = −0.32 e Å^−^^3^

### Special details

**Table:**

Geometry. All esds (except the esd in the dihedral angle between two l.s. planes) are estimated using the full covariance matrix. The cell esds are taken into account individually in the estimation of esds in distances, angles and torsion angles; correlations between esds in cell parameters are only used when they are defined by crystal symmetry. An approximate (isotropic) treatment of cell esds is used for estimating esds involving l.s. planes.

### Fractional atomic coordinates and isotropic or equivalent isotropic displacement parameters (Å^2^)

**Table:**

	*x*	*y*	*z*	*U*_iso_*/*U*_eq_	
Ir1	0.68717 (2)	0.18203 (2)	0.91417 (2)	0.02951 (4)	
Cl1	0.53215 (5)	0.24293 (5)	0.92683 (4)	0.04323 (17)	
P1	0.76225 (5)	0.28142 (5)	1.01468 (4)	0.03235 (16)	
P2	0.76117 (6)	0.48320 (5)	1.05116 (4)	0.03587 (17)	
N1	0.71230 (16)	0.27466 (14)	0.83889 (12)	0.0322 (5)	
N2	0.66418 (17)	0.07508 (14)	0.97668 (13)	0.0345 (5)	
C1	0.6505 (2)	0.33752 (19)	0.80720 (17)	0.0414 (7)	
H1	0.5898	0.3378	0.8167	0.050*	
C2	0.6752 (3)	0.4012 (2)	0.76128 (18)	0.0492 (8)	
H2	0.6313	0.4433	0.7395	0.059*	
C3	0.7646 (3)	0.4020 (2)	0.74807 (19)	0.0528 (9)	
H3	0.7830	0.4458	0.7187	0.063*	
C4	0.8269 (3)	0.3377 (2)	0.77858 (18)	0.0466 (8)	
H4	0.8880	0.3380	0.7700	0.056*	
C5	0.7996 (2)	0.27145 (18)	0.82245 (15)	0.0353 (6)	
C6	0.8552 (2)	0.19571 (18)	0.85264 (17)	0.0374 (7)	
C7	0.9445 (2)	0.1771 (2)	0.8388 (2)	0.0494 (8)	
H7	0.9729	0.2159	0.8111	0.059*	
C8	0.9905 (3)	0.1019 (2)	0.8659 (2)	0.0575 (9)	
H8	1.0506	0.0901	0.8577	0.069*	
C9	0.9467 (3)	0.0438 (2)	0.9055 (2)	0.0556 (9)	
H9	0.9766	−0.0082	0.9224	0.067*	
C10	0.8589 (2)	0.0618 (2)	0.92042 (18)	0.0449 (7)	
H10	0.8312	0.0217	0.9474	0.054*	
C11	0.8109 (2)	0.13846 (18)	0.89610 (15)	0.0338 (6)	
C12	0.7031 (2)	0.06046 (19)	1.05128 (17)	0.0469 (8)	
H12	0.7442	0.1017	1.0785	0.056*	
C13	0.6849 (3)	−0.0130 (2)	1.0891 (2)	0.0598 (10)	
H13	0.7127	−0.0209	1.1409	0.072*	
C14	0.6251 (3)	−0.0741 (2)	1.0492 (2)	0.0656 (11)	
H14	0.6112	−0.1240	1.0736	0.079*	
C15	0.5859 (3)	−0.0609 (2)	0.9727 (2)	0.0543 (9)	
H15	0.5446	−0.1019	0.9453	0.065*	
C16	0.6071 (2)	0.01343 (18)	0.93549 (17)	0.0373 (6)	
C17	0.5754 (2)	0.03060 (18)	0.85349 (16)	0.0366 (6)	
C18	0.5151 (2)	−0.0240 (2)	0.80214 (19)	0.0457 (7)	
H18	0.4890	−0.0725	0.8203	0.055*	
C19	0.4945 (2)	−0.0059 (2)	0.72503 (19)	0.0520 (8)	
H19	0.4546	−0.0424	0.6907	0.062*	
C20	0.5328 (2)	0.0662 (2)	0.69845 (18)	0.0488 (8)	
H20	0.5194	0.0778	0.6460	0.059*	
C21	0.5910 (2)	0.1216 (2)	0.74885 (16)	0.0409 (7)	
H21	0.6159	0.1701	0.7296	0.049*	
C22	0.61342 (19)	0.10635 (17)	0.82804 (15)	0.0324 (6)	
C23	0.8897 (2)	0.2899 (2)	1.02168 (16)	0.0381 (7)	
C24	0.9480 (2)	0.2230 (2)	1.0551 (2)	0.0531 (8)	
H24	0.9226	0.1784	1.0793	0.064*	
C25	1.0427 (3)	0.2207 (3)	1.0535 (2)	0.0675 (11)	
H25	1.0807	0.1751	1.0767	0.081*	
C26	1.0814 (3)	0.2856 (3)	1.0178 (3)	0.0708 (12)	
H26	1.1452	0.2839	1.0160	0.085*	
C27	1.0251 (3)	0.3526 (3)	0.9848 (2)	0.0621 (10)	
H27	1.0513	0.3969	0.9610	0.074*	
C28	0.9301 (2)	0.3557 (2)	0.98620 (18)	0.0460 (7)	
H28	0.8929	0.4019	0.9634	0.055*	
C29	0.7500 (2)	0.27335 (18)	1.11445 (16)	0.0388 (7)	
C30	0.6630 (3)	0.2510 (2)	1.12801 (19)	0.0529 (8)	
H30	0.6141	0.2342	1.0872	0.063*	
C31	0.6477 (3)	0.2534 (3)	1.2018 (2)	0.0694 (11)	
H31	0.5884	0.2392	1.2104	0.083*	
C32	0.7209 (4)	0.2769 (3)	1.2625 (2)	0.0792 (13)	
H32	0.7108	0.2784	1.3121	0.095*	
C33	0.8081 (4)	0.2980 (3)	1.2500 (2)	0.0743 (13)	
H33	0.8572	0.3133	1.2912	0.089*	
C34	0.8234 (3)	0.2965 (2)	1.17625 (19)	0.0551 (9)	
H34	0.8827	0.3110	1.1679	0.066*	
C35	0.7142 (2)	0.38963 (17)	0.98911 (16)	0.0381 (7)	
H35A	0.6463	0.3870	0.9857	0.046*	
H35B	0.7227	0.4023	0.9379	0.046*	
C36	0.6829 (2)	0.49398 (19)	1.11822 (17)	0.0395 (7)	
C37	0.5910 (3)	0.4657 (2)	1.1068 (2)	0.0625 (10)	
H37	0.5653	0.4332	1.0632	0.075*	
C38	0.5354 (3)	0.4842 (3)	1.1587 (3)	0.0831 (14)	
H38	0.4734	0.4636	1.1504	0.100*	
C39	0.5727 (3)	0.5334 (3)	1.2227 (2)	0.0721 (12)	
H39	0.5361	0.5458	1.2580	0.086*	
C40	0.6627 (3)	0.5638 (3)	1.2342 (2)	0.0699 (12)	
H40	0.6869	0.5988	1.2764	0.084*	
C41	0.7188 (3)	0.5429 (2)	1.18332 (19)	0.0565 (9)	
H41	0.7815	0.5619	1.1930	0.068*	
C42	0.7121 (2)	0.56941 (18)	0.98220 (17)	0.0383 (7)	
C43	0.6538 (3)	0.6345 (2)	0.9985 (2)	0.0520 (8)	
H43	0.6357	0.6344	1.0453	0.062*	
C44	0.6223 (3)	0.6995 (2)	0.9458 (2)	0.0659 (11)	
H44	0.5827	0.7424	0.9574	0.079*	
C45	0.6479 (3)	0.7019 (3)	0.8779 (2)	0.0673 (11)	
H45	0.6269	0.7467	0.8434	0.081*	
C46	0.7056 (3)	0.6376 (3)	0.8596 (2)	0.0594 (10)	
H46	0.7224	0.6381	0.8123	0.071*	
C47	0.7380 (2)	0.5729 (2)	0.91164 (18)	0.0468 (8)	
H47	0.7780	0.5306	0.8996	0.056*	

### Atomic displacement parameters (Å^2^)

**Table:**

	*U*^11^	*U*^22^	*U*^33^	*U*^12^	*U*^13^	*U*^23^
Ir1	0.03490 (7)	0.02715 (6)	0.02633 (6)	−0.00467 (4)	0.00662 (4)	−0.00016 (4)
Cl1	0.0386 (4)	0.0438 (4)	0.0497 (4)	−0.0044 (3)	0.0150 (3)	−0.0041 (3)
P1	0.0393 (4)	0.0284 (3)	0.0293 (4)	−0.0065 (3)	0.0075 (3)	−0.0017 (3)
P2	0.0401 (4)	0.0312 (4)	0.0358 (4)	−0.0056 (3)	0.0074 (3)	−0.0042 (3)
N1	0.0393 (13)	0.0303 (12)	0.0270 (11)	−0.0046 (10)	0.0074 (10)	0.0011 (9)
N2	0.0420 (14)	0.0295 (12)	0.0320 (12)	−0.0061 (10)	0.0085 (11)	0.0011 (10)
C1	0.0457 (18)	0.0408 (17)	0.0376 (16)	0.0004 (13)	0.0090 (14)	0.0047 (13)
C2	0.063 (2)	0.0413 (18)	0.0418 (17)	0.0032 (16)	0.0096 (16)	0.0115 (14)
C3	0.072 (2)	0.0458 (18)	0.0463 (19)	−0.0085 (17)	0.0247 (18)	0.0100 (15)
C4	0.053 (2)	0.0519 (19)	0.0410 (17)	−0.0136 (15)	0.0233 (16)	−0.0014 (14)
C5	0.0417 (17)	0.0379 (16)	0.0279 (13)	−0.0059 (12)	0.0110 (12)	−0.0045 (12)
C6	0.0394 (16)	0.0397 (16)	0.0334 (15)	−0.0049 (12)	0.0092 (13)	−0.0067 (12)
C7	0.0446 (19)	0.059 (2)	0.0473 (19)	−0.0043 (16)	0.0167 (16)	−0.0086 (16)
C8	0.047 (2)	0.069 (2)	0.057 (2)	0.0124 (18)	0.0130 (17)	−0.0148 (19)
C9	0.057 (2)	0.050 (2)	0.055 (2)	0.0152 (17)	0.0033 (18)	−0.0123 (17)
C10	0.0518 (19)	0.0372 (17)	0.0446 (17)	0.0032 (14)	0.0089 (15)	−0.0021 (13)
C11	0.0378 (16)	0.0347 (15)	0.0274 (13)	−0.0005 (12)	0.0039 (12)	−0.0074 (11)
C12	0.067 (2)	0.0370 (17)	0.0339 (15)	−0.0143 (15)	0.0066 (15)	0.0024 (13)
C13	0.088 (3)	0.048 (2)	0.0401 (18)	−0.0137 (19)	0.0080 (18)	0.0123 (15)
C14	0.090 (3)	0.0412 (19)	0.064 (2)	−0.0216 (19)	0.014 (2)	0.0167 (17)
C15	0.068 (2)	0.0337 (17)	0.057 (2)	−0.0173 (16)	0.0066 (18)	0.0020 (15)
C16	0.0403 (16)	0.0297 (14)	0.0410 (16)	−0.0047 (12)	0.0072 (13)	−0.0019 (12)
C17	0.0378 (16)	0.0342 (15)	0.0379 (15)	−0.0024 (12)	0.0084 (13)	−0.0078 (12)
C18	0.0412 (17)	0.0392 (17)	0.055 (2)	−0.0065 (13)	0.0065 (15)	−0.0099 (14)
C19	0.0437 (19)	0.054 (2)	0.051 (2)	−0.0006 (16)	−0.0045 (16)	−0.0211 (16)
C20	0.0482 (19)	0.059 (2)	0.0335 (16)	0.0087 (16)	−0.0022 (14)	−0.0101 (15)
C21	0.0437 (17)	0.0437 (17)	0.0348 (15)	0.0045 (13)	0.0075 (14)	0.0008 (13)
C22	0.0303 (14)	0.0344 (14)	0.0319 (14)	0.0002 (11)	0.0057 (12)	−0.0064 (11)
C23	0.0407 (17)	0.0386 (15)	0.0343 (15)	−0.0089 (13)	0.0071 (13)	−0.0098 (12)
C24	0.049 (2)	0.0446 (19)	0.062 (2)	−0.0051 (16)	0.0060 (17)	−0.0013 (16)
C25	0.046 (2)	0.065 (3)	0.083 (3)	0.0051 (19)	−0.001 (2)	−0.011 (2)
C26	0.042 (2)	0.079 (3)	0.093 (3)	−0.014 (2)	0.018 (2)	−0.027 (3)
C27	0.057 (2)	0.071 (3)	0.065 (2)	−0.027 (2)	0.026 (2)	−0.018 (2)
C28	0.0501 (19)	0.0464 (18)	0.0431 (17)	−0.0130 (15)	0.0137 (15)	−0.0107 (14)
C29	0.0549 (19)	0.0316 (15)	0.0297 (14)	−0.0039 (13)	0.0093 (14)	−0.0023 (12)
C30	0.058 (2)	0.060 (2)	0.0440 (18)	−0.0061 (17)	0.0199 (17)	−0.0005 (16)
C31	0.082 (3)	0.076 (3)	0.062 (2)	−0.003 (2)	0.039 (2)	0.001 (2)
C32	0.116 (4)	0.089 (3)	0.041 (2)	0.001 (3)	0.034 (3)	−0.004 (2)
C33	0.101 (4)	0.086 (3)	0.0325 (19)	−0.008 (3)	0.006 (2)	−0.0113 (19)
C34	0.064 (2)	0.059 (2)	0.0387 (18)	−0.0119 (18)	0.0044 (17)	−0.0041 (15)
C35	0.0490 (18)	0.0304 (14)	0.0326 (14)	−0.0048 (13)	0.0044 (13)	−0.0031 (12)
C36	0.0453 (18)	0.0354 (15)	0.0380 (16)	0.0017 (13)	0.0100 (14)	0.0043 (13)
C37	0.059 (2)	0.058 (2)	0.075 (3)	−0.0176 (18)	0.023 (2)	−0.0163 (19)
C38	0.063 (3)	0.078 (3)	0.126 (4)	−0.016 (2)	0.057 (3)	−0.009 (3)
C39	0.080 (3)	0.081 (3)	0.068 (3)	0.014 (2)	0.042 (2)	0.018 (2)
C40	0.075 (3)	0.094 (3)	0.0402 (19)	0.023 (2)	0.0102 (19)	−0.005 (2)
C41	0.048 (2)	0.077 (2)	0.0423 (18)	0.0061 (18)	0.0049 (16)	−0.0139 (17)
C42	0.0420 (17)	0.0328 (15)	0.0411 (16)	−0.0066 (12)	0.0113 (14)	−0.0014 (12)
C43	0.069 (2)	0.0363 (17)	0.057 (2)	0.0033 (16)	0.0286 (18)	0.0051 (15)
C44	0.076 (3)	0.049 (2)	0.079 (3)	0.0178 (19)	0.032 (2)	0.0156 (19)
C45	0.072 (3)	0.064 (2)	0.071 (3)	0.013 (2)	0.026 (2)	0.034 (2)
C46	0.063 (2)	0.067 (2)	0.051 (2)	−0.007 (2)	0.0206 (18)	0.0163 (18)
C47	0.050 (2)	0.0455 (18)	0.0477 (19)	−0.0042 (15)	0.0173 (16)	0.0002 (14)

### Geometric parameters (Å, º)

**Table:**

Ir1—C11	2.004 (3)	C20—C21	1.381 (4)
Ir1—C22	2.032 (3)	C20—H20	0.9300
Ir1—N1	2.051 (2)	C21—C22	1.397 (4)
Ir1—N2	2.062 (2)	C21—H21	0.9300
Ir1—P1	2.4241 (7)	C23—C24	1.381 (5)
Ir1—Cl1	2.4866 (8)	C23—C28	1.394 (4)
P1—C23	1.822 (3)	C24—C25	1.377 (5)
P1—C35	1.828 (3)	C24—H24	0.9300
P1—C29	1.834 (3)	C25—C26	1.373 (6)
P2—C36	1.830 (3)	C25—H25	0.9300
P2—C42	1.844 (3)	C26—C27	1.365 (6)
P2—C35	1.854 (3)	C26—H26	0.9300
N1—C1	1.354 (4)	C27—C28	1.380 (5)
N1—C5	1.360 (4)	C27—H27	0.9300
N2—C12	1.343 (4)	C28—H28	0.9300
N2—C16	1.362 (3)	C29—C30	1.376 (4)
C1—C2	1.378 (4)	C29—C34	1.394 (4)
C1—H1	0.9300	C30—C31	1.384 (5)
C2—C3	1.365 (5)	C30—H30	0.9300
C2—H2	0.9300	C31—C32	1.381 (6)
C3—C4	1.369 (5)	C31—H31	0.9300
C3—H3	0.9300	C32—C33	1.367 (6)
C4—C5	1.398 (4)	C32—H32	0.9300
C4—H4	0.9300	C33—C34	1.384 (5)
C5—C6	1.453 (4)	C33—H33	0.9300
C6—C7	1.397 (4)	C34—H34	0.9300
C6—C11	1.421 (4)	C35—H35A	0.9700
C7—C8	1.372 (5)	C35—H35B	0.9700
C7—H7	0.9300	C36—C37	1.369 (4)
C8—C9	1.382 (5)	C36—C41	1.385 (4)
C8—H8	0.9300	C37—C38	1.387 (5)
C9—C10	1.382 (5)	C37—H37	0.9300
C9—H9	0.9300	C38—C39	1.376 (6)
C10—C11	1.391 (4)	C38—H38	0.9300
C10—H10	0.9300	C39—C40	1.353 (6)
C12—C13	1.376 (4)	C39—H39	0.9300
C12—H12	0.9300	C40—C41	1.386 (5)
C13—C14	1.367 (5)	C40—H40	0.9300
C13—H13	0.9300	C41—H41	0.9300
C14—C15	1.370 (5)	C42—C43	1.384 (4)
C14—H14	0.9300	C42—C47	1.394 (4)
C15—C16	1.395 (4)	C43—C44	1.382 (5)
C15—H15	0.9300	C43—H43	0.9300
C16—C17	1.456 (4)	C44—C45	1.344 (5)
C17—C18	1.397 (4)	C44—H44	0.9300
C17—C22	1.411 (4)	C45—C46	1.384 (5)
C18—C19	1.370 (4)	C45—H45	0.9300
C18—H18	0.9300	C46—C47	1.372 (5)
C19—C20	1.376 (5)	C46—H46	0.9300
C19—H19	0.9300	C47—H47	0.9300
			
C11—Ir1—C22	91.12 (11)	C19—C20—C21	120.7 (3)
C11—Ir1—N1	80.35 (10)	C19—C20—H20	119.7
C22—Ir1—N1	92.62 (10)	C21—C20—H20	119.7
C11—Ir1—N2	94.40 (10)	C20—C21—C22	121.5 (3)
C22—Ir1—N2	80.07 (10)	C20—C21—H21	119.2
N1—Ir1—N2	170.97 (9)	C22—C21—H21	119.2
C11—Ir1—P1	93.75 (8)	C21—C22—C17	116.6 (3)
C22—Ir1—P1	174.57 (8)	C21—C22—Ir1	129.1 (2)
N1—Ir1—P1	85.83 (6)	C17—C22—Ir1	114.18 (19)
N2—Ir1—P1	101.93 (6)	C24—C23—C28	117.8 (3)
C11—Ir1—Cl1	175.37 (8)	C24—C23—P1	118.9 (2)
C22—Ir1—Cl1	87.54 (8)	C28—C23—P1	122.8 (3)
N1—Ir1—Cl1	95.27 (7)	C25—C24—C23	121.5 (3)
N2—Ir1—Cl1	89.74 (7)	C25—C24—H24	119.3
P1—Ir1—Cl1	87.41 (3)	C23—C24—H24	119.3
C23—P1—C35	105.78 (14)	C26—C25—C24	120.1 (4)
C23—P1—C29	104.84 (14)	C26—C25—H25	119.9
C35—P1—C29	100.97 (13)	C24—C25—H25	119.9
C23—P1—Ir1	111.90 (9)	C27—C26—C25	119.3 (4)
C35—P1—Ir1	108.25 (9)	C27—C26—H26	120.3
C29—P1—Ir1	123.42 (10)	C25—C26—H26	120.3
C36—P2—C42	99.77 (13)	C26—C27—C28	121.1 (4)
C36—P2—C35	105.37 (14)	C26—C27—H27	119.4
C42—P2—C35	97.50 (13)	C28—C27—H27	119.4
C1—N1—C5	119.5 (2)	C27—C28—C23	120.2 (3)
C1—N1—Ir1	125.2 (2)	C27—C28—H28	119.9
C5—N1—Ir1	115.23 (18)	C23—C28—H28	119.9
C12—N2—C16	119.0 (2)	C30—C29—C34	119.2 (3)
C12—N2—Ir1	126.07 (19)	C30—C29—P1	118.7 (2)
C16—N2—Ir1	114.83 (18)	C34—C29—P1	121.9 (3)
N1—C1—C2	121.7 (3)	C29—C30—C31	120.6 (4)
N1—C1—H1	119.1	C29—C30—H30	119.7
C2—C1—H1	119.1	C31—C30—H30	119.7
C3—C2—C1	119.5 (3)	C32—C31—C30	119.7 (4)
C3—C2—H2	120.3	C32—C31—H31	120.2
C1—C2—H2	120.3	C30—C31—H31	120.2
C2—C3—C4	119.2 (3)	C33—C32—C31	120.3 (4)
C2—C3—H3	120.4	C33—C32—H32	119.8
C4—C3—H3	120.4	C31—C32—H32	119.8
C3—C4—C5	120.7 (3)	C32—C33—C34	120.1 (4)
C3—C4—H4	119.6	C32—C33—H33	119.9
C5—C4—H4	119.6	C34—C33—H33	119.9
N1—C5—C4	119.1 (3)	C33—C34—C29	120.1 (4)
N1—C5—C6	114.3 (2)	C33—C34—H34	120.0
C4—C5—C6	126.5 (3)	C29—C34—H34	120.0
C7—C6—C11	121.1 (3)	P1—C35—P2	119.78 (16)
C7—C6—C5	123.8 (3)	P1—C35—H35A	107.4
C11—C6—C5	115.1 (3)	P2—C35—H35A	107.4
C8—C7—C6	120.4 (3)	P1—C35—H35B	107.4
C8—C7—H7	119.8	P2—C35—H35B	107.4
C6—C7—H7	119.8	H35A—C35—H35B	106.9
C7—C8—C9	119.2 (3)	C37—C36—C41	117.8 (3)
C7—C8—H8	120.4	C37—C36—P2	126.5 (3)
C9—C8—H8	120.4	C41—C36—P2	115.4 (2)
C8—C9—C10	121.0 (3)	C36—C37—C38	121.6 (4)
C8—C9—H9	119.5	C36—C37—H37	119.2
C10—C9—H9	119.5	C38—C37—H37	119.2
C9—C10—C11	121.7 (3)	C39—C38—C37	119.4 (4)
C9—C10—H10	119.1	C39—C38—H38	120.3
C11—C10—H10	119.1	C37—C38—H38	120.3
C10—C11—C6	116.5 (3)	C40—C39—C38	120.0 (4)
C10—C11—Ir1	129.5 (2)	C40—C39—H39	120.0
C6—C11—Ir1	114.0 (2)	C38—C39—H39	120.0
N2—C12—C13	122.8 (3)	C39—C40—C41	120.3 (4)
N2—C12—H12	118.6	C39—C40—H40	119.9
C13—C12—H12	118.6	C41—C40—H40	119.9
C14—C13—C12	118.8 (3)	C36—C41—C40	120.9 (4)
C14—C13—H13	120.6	C36—C41—H41	119.6
C12—C13—H13	120.6	C40—C41—H41	119.6
C13—C14—C15	119.2 (3)	C43—C42—C47	117.5 (3)
C13—C14—H14	120.4	C43—C42—P2	123.0 (2)
C15—C14—H14	120.4	C47—C42—P2	119.4 (2)
C14—C15—C16	120.8 (3)	C44—C43—C42	120.4 (3)
C14—C15—H15	119.6	C44—C43—H43	119.8
C16—C15—H15	119.6	C42—C43—H43	119.8
N2—C16—C15	119.3 (3)	C45—C44—C43	121.3 (4)
N2—C16—C17	115.5 (2)	C45—C44—H44	119.3
C15—C16—C17	125.2 (3)	C43—C44—H44	119.3
C18—C17—C22	121.3 (3)	C44—C45—C46	119.7 (3)
C18—C17—C16	123.9 (3)	C44—C45—H45	120.2
C22—C17—C16	114.7 (2)	C46—C45—H45	120.2
C19—C18—C17	119.9 (3)	C47—C46—C45	119.7 (3)
C19—C18—H18	120.0	C47—C46—H46	120.2
C17—C18—H18	120.0	C45—C46—H46	120.2
C18—C19—C20	119.9 (3)	C46—C47—C42	121.3 (3)
C18—C19—H19	120.1	C46—C47—H47	119.3
C20—C19—H19	120.1	C42—C47—H47	119.3
			
C5—N1—C1—C2	−3.1 (4)	C35—P1—C23—C28	−21.3 (3)
Ir1—N1—C1—C2	175.3 (2)	C29—P1—C23—C28	−127.5 (2)
N1—C1—C2—C3	−1.0 (5)	Ir1—P1—C23—C28	96.4 (2)
C1—C2—C3—C4	2.3 (5)	C28—C23—C24—C25	−0.2 (5)
C2—C3—C4—C5	0.3 (5)	P1—C23—C24—C25	171.8 (3)
C1—N1—C5—C4	5.6 (4)	C23—C24—C25—C26	−0.4 (6)
Ir1—N1—C5—C4	−172.9 (2)	C24—C25—C26—C27	0.8 (6)
C1—N1—C5—C6	−173.3 (3)	C25—C26—C27—C28	−0.6 (6)
Ir1—N1—C5—C6	8.1 (3)	C26—C27—C28—C23	0.0 (5)
C3—C4—C5—N1	−4.3 (4)	C24—C23—C28—C27	0.4 (5)
C3—C4—C5—C6	174.5 (3)	P1—C23—C28—C27	−171.3 (2)
N1—C5—C6—C7	176.2 (3)	C23—P1—C29—C30	−168.8 (3)
C4—C5—C6—C7	−2.6 (5)	C35—P1—C29—C30	81.4 (3)
N1—C5—C6—C11	−1.6 (4)	Ir1—P1—C29—C30	−39.2 (3)
C4—C5—C6—C11	179.6 (3)	C23—P1—C29—C34	17.0 (3)
C11—C6—C7—C8	1.2 (5)	C35—P1—C29—C34	−92.8 (3)
C5—C6—C7—C8	−176.5 (3)	Ir1—P1—C29—C34	146.5 (2)
C6—C7—C8—C9	1.5 (5)	C34—C29—C30—C31	1.5 (5)
C7—C8—C9—C10	−2.3 (5)	P1—C29—C30—C31	−172.9 (3)
C8—C9—C10—C11	0.3 (5)	C29—C30—C31—C32	−1.2 (6)
C9—C10—C11—C6	2.3 (4)	C30—C31—C32—C33	0.2 (7)
C9—C10—C11—Ir1	−177.0 (2)	C31—C32—C33—C34	0.5 (7)
C7—C6—C11—C10	−3.1 (4)	C32—C33—C34—C29	−0.2 (6)
C5—C6—C11—C10	174.9 (3)	C30—C29—C34—C33	−0.8 (5)
C7—C6—C11—Ir1	176.3 (2)	P1—C29—C34—C33	173.4 (3)
C5—C6—C11—Ir1	−5.8 (3)	C23—P1—C35—P2	−56.6 (2)
C16—N2—C12—C13	−2.7 (5)	C29—P1—C35—P2	52.4 (2)
Ir1—N2—C12—C13	−179.5 (3)	Ir1—P1—C35—P2	−176.66 (14)
N2—C12—C13—C14	0.4 (6)	C36—P2—C35—P1	−93.0 (2)
C12—C13—C14—C15	0.6 (6)	C42—P2—C35—P1	164.65 (18)
C13—C14—C15—C16	0.6 (6)	C42—P2—C36—C37	76.6 (3)
C12—N2—C16—C15	3.9 (4)	C35—P2—C36—C37	−24.0 (3)
Ir1—N2—C16—C15	−179.0 (2)	C42—P2—C36—C41	−97.0 (3)
C12—N2—C16—C17	−174.2 (3)	C35—P2—C36—C41	162.4 (2)
Ir1—N2—C16—C17	3.0 (3)	C41—C36—C37—C38	−0.6 (5)
C14—C15—C16—N2	−2.9 (5)	P2—C36—C37—C38	−174.1 (3)
C14—C15—C16—C17	174.9 (3)	C36—C37—C38—C39	1.0 (7)
N2—C16—C17—C18	−178.7 (3)	C37—C38—C39—C40	0.5 (7)
C15—C16—C17—C18	3.4 (5)	C38—C39—C40—C41	−2.4 (6)
N2—C16—C17—C22	3.7 (4)	C37—C36—C41—C40	−1.3 (5)
C15—C16—C17—C22	−174.2 (3)	P2—C36—C41—C40	172.9 (3)
C22—C17—C18—C19	2.3 (5)	C39—C40—C41—C36	2.8 (6)
C16—C17—C18—C19	−175.2 (3)	C36—P2—C42—C43	16.7 (3)
C17—C18—C19—C20	−0.4 (5)	C35—P2—C42—C43	123.8 (3)
C18—C19—C20—C21	−0.9 (5)	C36—P2—C42—C47	−166.7 (2)
C19—C20—C21—C22	0.4 (5)	C35—P2—C42—C47	−59.6 (3)
C20—C21—C22—C17	1.4 (4)	C47—C42—C43—C44	0.6 (5)
C20—C21—C22—Ir1	−174.3 (2)	P2—C42—C43—C44	177.3 (3)
C18—C17—C22—C21	−2.7 (4)	C42—C43—C44—C45	−0.6 (6)
C16—C17—C22—C21	175.0 (3)	C43—C44—C45—C46	1.0 (7)
C18—C17—C22—Ir1	173.6 (2)	C44—C45—C46—C47	−1.5 (6)
C16—C17—C22—Ir1	−8.7 (3)	C45—C46—C47—C42	1.5 (5)
C35—P1—C23—C24	167.1 (2)	C43—C42—C47—C46	−1.1 (5)
C29—P1—C23—C24	60.9 (3)	P2—C42—C47—C46	−177.9 (3)
Ir1—P1—C23—C24	−75.2 (3)		

### Hydrogen-bond geometry (Å, º)

*Cg*6, *Cg*8 and *Cg*10 are the centroids of the C17–C22, C29–C34 and C42–C47 rings, respectively.

**Table:**

*D*—H···*A*	*D*—H	H···*A*	*D*···*A*	*D*—H···*A*
C1—H1···Cl1	0.93	2.73	3.357 (3)	126
C14—H14···Cl1^i^	0.93	2.77	3.548 (4)	142
C30—H30···Cl1	0.93	2.84	3.664 (4)	148
C35—H35*A*···Cl1	0.97	2.83	3.460 (3)	124
C3—H3···*Cg*6^ii^	0.93	2.83	3.572 (4)	137
C26—H26···*Cg*10^iii^	0.93	2.79	3.575 (5)	143
C38—H38···C10^iii^	0.93	2.81	3.659 (5)	153
C40—H40···*Cg*8^iv^	0.93	2.95	3.711 (5)	140

Symmetry codes: (i) −*x*+1, −*y*, −*z*+2; (ii) −*x*+3/2, *y*+1/2, −*z*+3/2; (iii) −*x*+2, −*y*+1, −*z*+2; (iv) −*x*+3/2, *y*+1/2, −*z*+5/2.
